# Epidemiological investigation and drug resistance of *Eimeria* species in Korean chicken farms

**DOI:** 10.1186/s12917-022-03369-3

**Published:** 2022-07-14

**Authors:** Rochelle A. Flores, Binh T. Nguyen, Paula Leona T. Cammayo, Tuấn Cường Võ, Haung Naw, Suk Kim, Woo H. Kim, Byoung-Kuk Na, Wongi Min

**Affiliations:** 1 College of Veterinary Medicine & Institute of Animal Medicine, Gyeongsang National University, Jinju, 52828 Republic of Korea; 2 Department of Parasitology and Tropical Medicine, Department of Convergence Medical Science, and Institute of Health Sciences, Gyeongsang National University College of Medicine, Jinju, 52727 Republic of Korea

**Keywords:** Anticoccidial drug resistance, Chickens, Coccidiosis, Epidemiological investigation, Korea

## Abstract

**Background:**

Coccidiosis is a poultry disease that occurs worldwide and is caused by *Eimeria* species. The infection is associated with reduced feed efficiency, body weight gain, and egg production. This study aimed to investigate the current status of coccidiosis and anticoccidial resistance to anticoccidial drugs used as part of control strategies for this disease in Korean chicken farms.

**Results:**

An overall prevalence of 75% (291/388) was found. Positive farms contained several *Eimeria* species (mean = 4.2). Of the positive samples, *E. acervulina* (98.6%)*, E. maxima* (84.8%)*, *and *E. tenella* (82.8%) were the most prevalent species. Compared with cage-fed chickens, broilers and native chickens reared in free-range management were more at risk of acquiring an *Eimeria* infection. Sensitivities to six anticoccidial drugs (clopidol, diclazuril, maduramycin, monensin, salinomycin, and toltrazuril) were tested using nine field samples. Compared with untreated healthy control chickens, the body weight gains of infected chickens and treated/infected chickens were significantly reduced in all groups. Fecal oocyst shedding was significantly reduced in four clopidol-treated/infected groups, three diclazuril-treated/infected groups, two toltrazuril-treated/infected groups, one monensin-treated/infected group, and one salinomycin-treated/infected group, compared with the respective untreated/infected control groups. Intestinal lesion scores were also reduced in three clopidol-treated/infected groups, one monensin-treated/infected group, and one toltrazuril-treated/infected group. However, an overall assessment using the anticoccidial index, percent optimum anticoccidial activity, relative oocyst production, and reduced lesion score index found that all field samples had strong resistance to all tested anticoccidial drugs.

**Conclusion:**

The results of this large-scale epidemiological investigation and anticoccidial sensitivity testing showed a high prevalence of coccidiosis and the presence of severe drug resistant *Eimeria* species in the field. These findings will be useful for optimizing the control of coccidiosis in the poultry industry.

**Supplementary Information:**

The online version contains supplementary material available at 10.1186/s12917-022-03369-3.

## Background

The meat and eggs from poultry are significant sources of protein. Chickens are the most essential poultry species globally; they constitute about 90% of the poultry population worldwide [1]. Coccidiosis is a major cause of immunosuppression in poultry. It is identified by the American Association of Avian Pathologists as one of the top diseases of concern affecting broiler and layer farms [2]. This disease occurs worldwide and is caused by any one of seven *Eimeria* species. *Eimeria* is an intracellular parasite that invades the intestinal epithelial cells of chickens. Infection that progresses to disease causes mortality and production losses associated with reduced feed efficiency, body weight gain, and egg production in affected chickens [3–5].

Coccidiosis is endemic in most tropical and subtropical regions where farm management practices mostly include use of deep litter that creates environmental conditions that favor year-round propagation of *Eimeria* species. In practice, increasing use of intensive farming systems and the associated high stocking densities on farms increases the likelihood of disease persistence. Based on 2016 prices, the estimated global cost of coccidiosis control is £10.36 billion [6]. Consistent year-by-year financial pressures on the poultry industry are linked to prophylactic use of anticoccidial drugs (e.g., ionophores and synthetic chemicals) in feed or water [7]. However, development of drug resistance over time in different parts of the world have reduced efficacy of anticoccidials [8, 9]. To address the growing consumer demand for antibiotic-free poultry products and appearance of drug-resistant strains, regulations implemented to ban the use of antibiotics in food-producing animals, and natural dietary supplements and probiotics are being used as alternative coccidiosis control strategies [8, 10–13]. Low-dose virulent or attenuated vaccine strains of *Eimeria* are also used to control and prevent infection [4, 14–17]. However, there is little cross-protection between *Eimeria* species, and there are between-species differences in susceptibility to anticoccidial drugs [18, 19]. Therefore, it is crucial to accurately identify *Eimeria* species to select more effective vaccines and control measures.

In our previous report in 2010, the prevalence of coccidiosis was 78.7% in fecal samples from 356 poultry farms. By using a species-specific PCR diagnostic method based on the internal transcribed spacer 1 sequence, *E. acervulina* and *E. tenella* were the most common in the investigated farms with 87.5% and 62.5%, respectively, followed by *E. brunetti* and *E. praecox* (59.3%, and 37.5%, respectively). The other species such as *E. maxima*, *E. necatrix,* and *E. mitis* had a prevalence of 31.3% [20]. There have also been studies of the use of various natural substances to control coccidiosis, such as aloe vera [13], berberine [21], galla rhois [22] and green tea [23]. The objective of this study is to provide an up-to-date status of coccidiosis in Korea by investigating the occurrence of *Eimeria* infection in poultry farms and comparing the resistance of field *Eimeria* species to commercially available anticoccidial drugs. The resulting epidemiological data can be used to develop guidelines for control measures that are more appropriate to the current status of this disease.

## Results

### Prevalence and distribution of coccidiosis

**Fig. 1:**
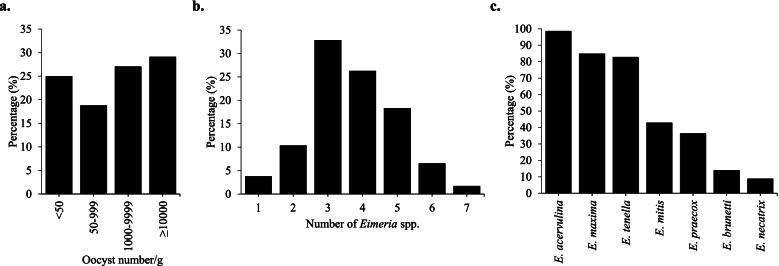
Prevalence and distribution of coccidiosis in Korean chicken farms. **A** Frequency of the number of oocysts per gram of fecal samples. **B** Specific rate and number of *Eimeria* species present in positive sample. **C** Distribution of *Eimeria* species in samples

Of the fecal samples collected from 388 chicken farms, 291 were positive for coccidian oocysts (overall prevalence = 75%). The specific rates (prevalence rates) in chicken types were determined for broilers (84.5%), native chickens (81.48%), and breeder and laying hens (33.3–42.6%). Similarly, prevalence rates in the management types of free-range chickens (83.0%) and caged chickens (41.3%) were identified (Table 1). The numbers of oocysts in fecal samples were < 50/g (25%), 50–999/g (18.8%), 1,000–9,999/g (27.1%), and ≥ 10,000 (29.1%) (Fig. 1A). The 90.2% and 96.4% of fecal samples containing ≥ 10,000 oocysts/g were from broilers and free-range chickens, respectively (Table 2). Positive fecal samples included multiple *Eimeria* species (mean = 4.2). There were 3 to approximately 5 *Eimeria* species in 77.5% of the positive samples (Fig. 1B). The most prevalent *Eimeria* species were *E. acervulina* (98.6%), *E. maxima* (84.8%), and *E. tenella* (82.8%), followed by *E. mitis* (42.9%), *E. praecox* (36.4%)*, E. brunetti* (14%), and *E. necatrix* (8.8%) (Fig. 1C).

**Table 1:** Distribution and risk factor analysis of avian coccidiosis

	**Total cases (%)**	**Cases of coccidiosis (%)**	**Specific Rates (%)**	**OR**	**95% CI**	***P* value**	
**Type of Chickens**							
Broiler	278 (71.65)	235 (80.76)	84.53	7.36	4.028–13.24	< 0.0001	***
Native chicken	27 (6.96)	22 (7.56)	81.48	5.92	2.058–15.56	< 0.001	***
Broiler breeder	12 (3.09)	4 (1.37)	33.33	0.67	0.2085–2.296	0.7502	NS
Layer Breeder	10 (2.58)	4 (1.37)	40.00	0.90	0.2646–3.182	> 0.999	NS
Lay hen	61 (15.72)	26 (8.93)	42.62	Reference			
TOTAL	388 (100)	291 (100)	75.00				
**Type of Management**							
Free-range	313 (80.67)	260 (89.35)	83.07	6.963	4.089–11.73	< 0.0001	***
Caged	75 (19.33)	31 (10.65)	41.33	Reference			
TOTAL	388 (100)	291 (100)	75.00				

*OR* Odds Ratio, *CI* Confidence Interval, *NS* Not significant

^***^P < 0.001

**Table 2:** Risk analysis with oocyst concentrations ≥ 10,000 oocyst numbers per gram of feces in individual sample

	**Total cases (%)**	**Cases of over 10,000 oocyst/g (%)**	**Specific Rates (%)**	**OR**	**95% CI**	***P* value**	
**Type of Chickens**							
Broiler	278 (71.65)	102 (90.27)	36.69	8.26	3.115–21.78	< 0.0001	***
Native chicken	27 (6.96)	7 (6.19)	25.93	4.99	1.319–16.17	0.0303	*
Broiler breeder	12 (3.09)	0 (0)	0.00	0.00	0.00–5.698	> 0.999	NS
Layer Breeder	10 (2.58)	0 (0)	0.00	0.00	0.00–7.060	> 0.999	NS
Lay hen	61 (15.72)	4 (3.54)	6.56	Reference			
TOTAL	388 (100)	113 (100)	29.12				
**Type of Management**							
Free-ranged	313 (80.67)	109 (96.46)	34.82	9.484	3.449–24.85	< 0.0001	***
Caged	75 (19.33)	4 (3.54)	5.33	Reference			
TOTAL	388 (100)	113 (100)	29.12				

*OR* Odds Ratio, *CI* Confidence Interval, *NS* Not significant

^*^ *P* < 0.05*,* ***P < 0.001

### Risk factor analysis and coccidiosis infection status

Based on the specific rates of prevalence in chicken types (Table 1), broilers and native chickens were about 7.3 and 5.9 times, respectively, more at risk than laying hens. Free-range chickens were about 6.9 times more at risk than caged chickens (Table 1). With fecal samples containing ≥ 10,000 oocysts/g, broilers and native chickens were about 8.2 and 4.9 times, respectively, more at risk than laying hens. Free-range chickens were about 9.4 times more at risk than caged chickens (Table 2).

**Fig. 2:**
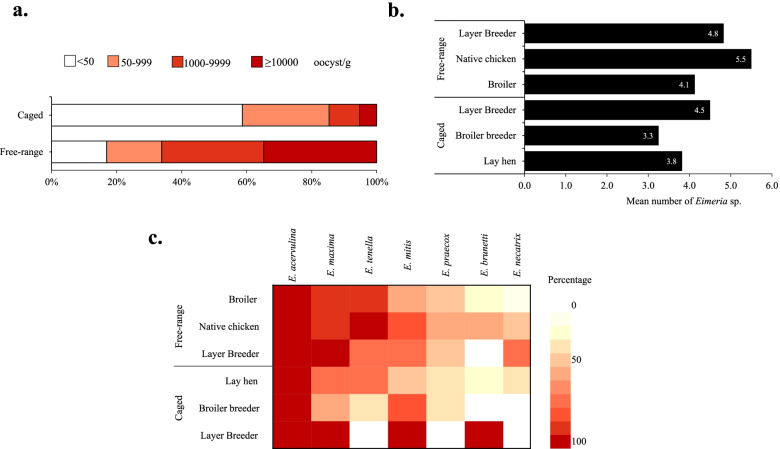
Status of coccidiosis based on level of infection and *Eimeria* species. **A** Level of infection based on the number of oocysts per gram of fecal samples. **B** Mean of numbers of *Eimeria* species present in positive samples. **C** Species-specific distribution of *Eimeria* species based on the source of fecal samples

Analysis of fecal oocyst concentration indicated that most samples had < 50 oocysts/g (58.6%) in fecal samples obtained from caged chickens; most samples collected from free-range chickens had ≥ 10,000 oocysts/g (34.8%) (Fig. 2A). The mean number of *Eimeria* species was not different between free-range (4.8 ± 0.7) and caged chickens (3.9 ± 0.6) (Fig. 2B). In general, regardless of chicken breed and management type, *E. acervulina* and *E. maxima* were the most prevalent *Eimeria* species (Fig. 2C).

### Anticoccidial sensitivity test of field samples

**Fig. 3:**
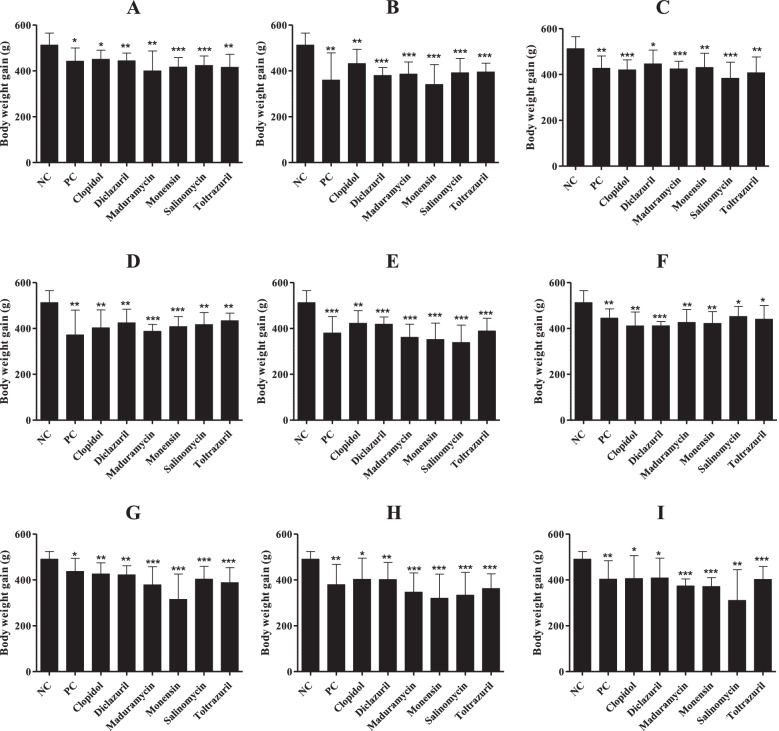
Comparison of body weight gain in field sample-infected chickens. Five-day-old ROSS 308 female chickens were orally infected with 3 × 10^4^ sporulated oocysts of 9 different field samples (**A**–**I**)*.* Body weight gain (*n* = 20) was measured at 9 days after infection. * *P* < 0.05, ** P < 0.01, and ***P < 0.001 indicate significant differences compared to untreated and healthy group (NC). The results represent mean ± SE values. NC, negative control; PC, untreated and infected group as a positive control; **A**-**I**, farm samples

**Fig. 4:**
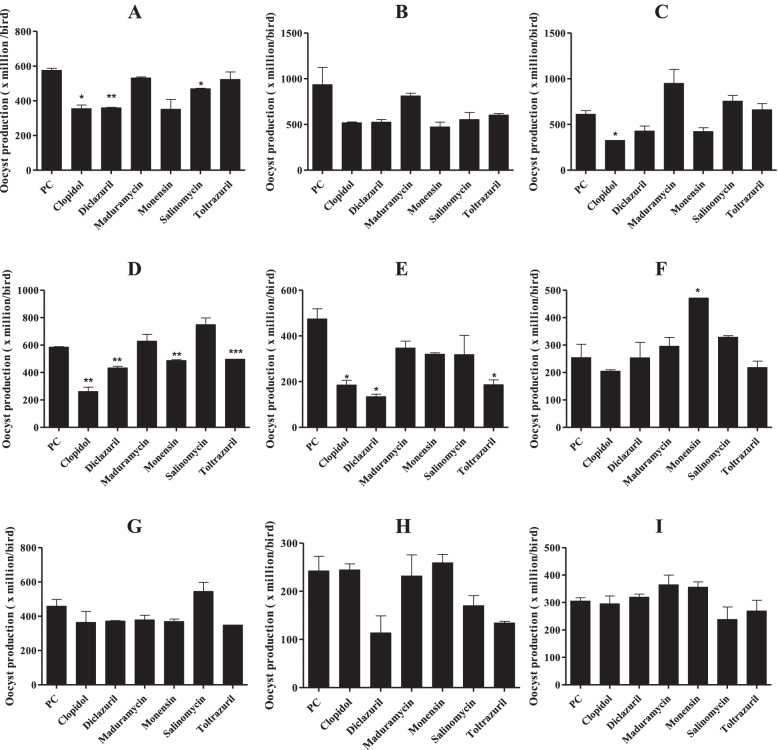
Comparison of oocyst numbers in field sample-infected chickens. Five-day-old ROSS 308 female chickens were orally infected with 3 × 10^4^ sporulated oocysts of 9 different field samples (**A**–**I**)*.* Production of oocysts per group (*n* = 20) was obtained from the fecal samples collected from days 6 to 9 post-infection. * *P* < 0.05, ** P < 0.01, and ***P < 0.001 indicate significant differences compared to untreated and infected group (PC). The results represent mean ± SE values. NC, negative control; PC, untreated and infected group as a positive control; **A**-**I**, farm samples

**Fig. 5:**
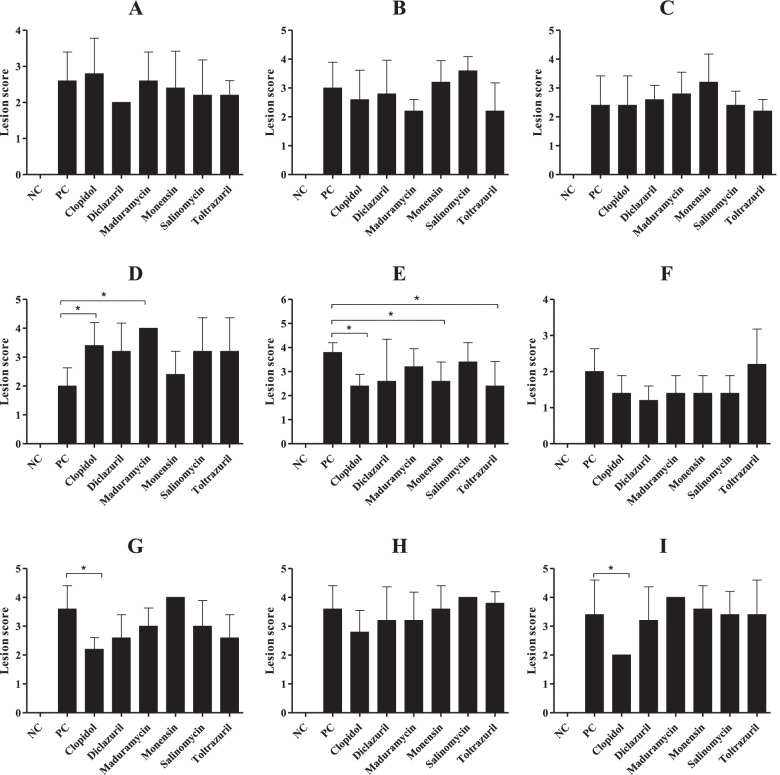
Comparison of intestinal lesion scores in field sample-infected chickens. Five-day-old ROSS 308 female chickens were orally infected with 3 × 10^4^ sporulated oocysts of 9 different field samples (**A**–**I**)*.* At day 7 post-infection, five chickens from each group were randomly selected for intestinal lesion scoring. Lesions scores range from 0–4, following the Johnson and Reid [24] scoring technique. * *P* < 0.05 indicates significant difference compared to untreated and infected group (PC). Results represent mean ± SE values. NC, negative control; PC, untreated and infected group as a positive control; **A**-**I**, farm samples

Nine field samples (A–I) collected from different provinces were used for the anticoccidial experiment against six different drugs. Parameters such as body weight gain, oocyst production, and lesion score were measured (Figs. 3, 4 and 5). The nine field samples included the most prevalent *Eimeria* species (*E. acervulina*, *E. maxima*, and *E. tenella*) with 0 to 3 different *Eimeria* species depending on the sample (Additional file 1).

Compared with untreated healthy control (NC) animals, body weight gains of untreated and infected chickens (PC) and treated/infected chickens were significantly reduced in all groups. There was no difference in body weight gain between PC and treated/infected chickens (Fig. 3). Fecal oocyst shedding was significantly reduced in 4 clopidol-treated/infected groups, 3 diclazuril-treated/infected groups, 2 toltrazuril-treated/infected groups, 1 monensin-treated/infected group, and 1 salinomycin-treated/infected group, compared with their respective PC groups. No oocysts were found in the NC groups (Fig. 4). Intestinal lesion scores were reduced in 3 clopidol-treated/infected groups, 1 monensin-treated/infected group, and 1 toltrazuril-treated/infected group. No lesion scores were found in the NC groups (Fig. 5).

Next, the four anticoccidial indexes, the ACI, POAA, ROP and RLS index, were evaluated with body weight gain, oocyst production and the lesion scores used above and survival rates (Additional file 2). The results for the ACI, POAA, ROP, and RLS values indicated that all field samples were resistant to all anticoccidial drugs (Additional files 3, 4, 5 and 6). Taken together, the results indicated that all field samples had severe resistance to all anticoccidial drugs tested (Table 3).

**Table 3:** Overall assessment of the field samples to anticoccidial drugs

Treatment	Farm samples								
	A	B	C	D	E	F	G	H	I
Clopidol	4	4	4	4	4	3	4	4	4
Diclazuril	4	4	4	4	4	4	4	4	4
Maduramycin	3	4	4	4	4	4	4	4	4
Monensin	4	4	4	4	4	4	3	4	3
Salinomycin	4	4	4	4	4	4	4	4	3
Toltrazuril	4	4	4	4	4	4	4	4	4

Anticoccidial drug resistance was evaluated using the anticoccidial index (ACI), percent optimum anticoccidial activity (POAA), relative oocyst production (ROP), and reduced lesion score (RLS). After giving each of the values of resistance (1) and non-resistance (0) to the four methods used, the overall evaluation was calculated by adding them together. Interpretation of results; no drug resistance (0), slightly drug resistant (1), moderately drug resistant (2) and severely drug resistant (3 or 4). A-I, farm samples

## Discussion

Worldwide, coccidiosis is an economically significant disease in the poultry sector. Therefore, regular epidemiological monitoring of *Eimeria* species present in the field and up-to-date status of resistance to treatments are vital for the best selection of control and prevention strategies. Overall, this study found that the current prevalence of coccidiosis in Korea is 75% (291 of 388 farms). Compared with our laboratory's previous epidemiological findings of 78.7% (280 of the 356 farms) in 2010, this finding was lower, but generally higher than the coccidiosis prevalence values reported in South Ethiopia, Pakistan, Nigeria, Iran, India, Turkey, and Algeria of 20.10%, 23.8%, 31.8%, 35.2%, 39.58%, 54.3%, and 63.26%, respectively [1, 20, 25–30]. Factors including control methods used, timing of sampling, animal husbandry, and geographical variation can account for differences in prevalence, compared with previous studies [31]. But, comparing the previous results for South Korea to this study suggests there was little progress in control for the past 10 years.

In this study, the high prevalence of coccidiosis based on poultry type and management type was associated with broilers (84.5%) and native chickens (81.48%), particularly those raised using the free-range (83.0%) management (Table 1). While these findings are contrary to the results of a study in Nigeria, which found the layer chickens have a higher prevalence rate of coccidial infection than broilers, they are consistent with the findings in Jordan and Iran poultry industries [32–34]. The previous studies found that the high prevalence values of coccidiosis in broilers are associated with deep-litter management with high stocking densities and poor farm management practices [33, 34]. In Korea, free-range management includes use of deep litter with high stocking densities. This study found a high degree of association between disease occurrence and management type; chickens raised using a free-range type of management had a higher coccidiosis rate. The fecal–oral route is the mode of transmission of the infective state of *Eimeria* species (sporulated oocysts) [35]. Use of deep litter provides conditions that promote oocyst accumulation, sporulation, and persistence in the environment. Consequently, the rate of exposure or contact of chickens to *Eimeria* species increases via the contaminated litter or fomites. Coccidiosis prevalence in birds raised in wire cages is lower because fecal–oral transmission is essentially stopped or reduced [36].

To reflect current infection status in the field, collected fecal samples were further assessed by determining OPG values, the numbers of *Eimeria* species present in a single positive sample, and identification of the specific *Eimeria* species in fecal samples. First, monitoring oocyst number in fecal samples provides an index of the degree of infection and the parasite's rate of reproduction in the intestines [37]. Although the OPG values in litter varied widely between samples, high numbers of samples with oocyst numbers ≥ 10,000/g were consistently found in broilers and free-range chickens; 56.2% of the fecal samples contained ≥ 1,000 oocysts/g (Fig. 1A). This result suggested that there was a considerable amount of oocyst output in the field and that oocysts would continue to accumulate, sporulate, and infect more birds. It is interesting to note that even a mild infection of about 50 *E. tenella* or *E. brunetti* oocysts can yield several hundred thousand oocysts [38]. Although this OPG monitoring revealed estimates of infection levels, it did not collectively represent the course of infection in the flock; it only provided an estimate of the numbers of oocysts present at the time of sampling. Long and Rowell found that oocyst numbers generally peak at 4–5 weeks after the introduction of birds [39]. Thus, further studies of the course of infection should include flock age and information about litter recycling at the time of sampling.

Each species of *Eimeria* that infects poultry has a preferred site of infection in the bird's gastrointestinal tract. Lesions can be mild or severe, depending on the magnitude of infection. While some studies found single-species infections in flocks, coccidiosis mainly results from a mix of *Eimeria* species that parasitize different areas of the intestine [12, 40]. Consistent with this finding, the mean number of *Eimeria* present in a single sample was determined to demonstrate the degree of infection in the field and the *Eimeria* species present on the samples were identified to discriminate proper measures for control, as some *Eimeria* species are predisposing factors to necrotic enteritis [41]. This study found mixed populations of *Eimeria* species (mean = 4.2); 77.5% of the positive samples contained 3 to 5 *Eimeria* species in one fecal sample (Fig. 1B). This high prevalence of mixed infection in samples was consistent with findings of > 80% in Norway, 55% in Nigeria, and 54.28% in Algeria [25, 31, 42].

RT-PCR analysis of the positive fecal samples detected all *Eimeria* species in the fecal samples. *Eimeria acervulina* (98.6%), *E. maxima* (84.8%), and *E. tenella* (82.8%) were the most prevalent species. The finding that *E. acervulina* was the predominant *Eimeria* species was consistent with previous findings in South Korea, Southwestern Nigeria, and Romania [9, 20, 43]. However, it was not consistent with the results of studies of free-range chickens in Tunisia and broiler farms in Northern Jordan, where the prevalence of *E. acervulina* are 1.5% and 3%, respectively [33, 44]. The high prevalence of *E. acervulina* and *E. tenella* are likely attributable to the high oocyst production rates of both species while the detected high prevalence of *E. maxima* can be attributed to the high potential of this species to extensively affect a large portion of the intestine, compared with other species [45]. In this study, the findings for *E. brunetti* (14%) and *E. necatrix* (8.8%) were consistent with the finding of a low species prevalence in China, but were not consistent with findings in South Korea and India [19, 46, 47]. These differences may be associated with alterations in the population dynamics of the parasite brought about by the different reproductive potential of each *Eimeria* species [45]. Also, *E. brunetti* and *E. necatrix* are more common in older birds [48].

Compared with the results of a previous study in South Korea performed 10 years ago, this study found that the prevalence of coccidiosis remains high. The mean number of *Eimeria* species per sample increased from 3.4 to 4.2, accompanied by evidence of a change in population dynamics of the infecting species present in the field (Fig. 1). The varying resistance of *Eimeria* species to anticoccidials used in other countries is well-documented [42, 49, 50]. However, in South Korea, the drug-resistant strains of *Eimeria* species are poorly understood and documented. We also evaluated the sensitivities of nine field samples to anticoccidials. We used six anticoccidial drugs used in the field (clopidol, diclazuril, maduramycin, monensin, salinomycin, and toltrazuril). Parameters such as body weight gain, intestinal lesion scores, and oocyst production along with mortality rates were assessed. Treatments are considered protective when birds maintain weight gain and lesion scores are zero or at a low number [51]. Our trials found that all infected chickens had reduced body weight gain (Fig. 3), which is a clinical manifestation of coccidiosis. But, while oocyst production (Fig. 4) and lesion scores (Fig. 5) were reduced in some medicated groups, limited levels of reduction in lesion scores and oocyst production were found in most medicated groups. In general, assessment of drug efficacy based on a single parameter may result in inconsistent findings [52]. Thus, we used a standard method for an overall assessment of drug sensitivity (i.e., the ACI, POAA, ROP, and RLS indices). Overall, the results indicated that all field samples collected in this study were severely drug resistant to all anticoccidials evaluated, but particularly to diclazuril, maduramycin, and toltrazuril. Likewise, varying levels of drug resistance to clopidol [53], diclazuril [49, 54], maduramycin [55], monensin [56, 57], salinomycin [56–58], and toltrazuril [42, 49] were previously reported. Similar to the findings in this study, presence of field isolates with multi-drug resistance to anticoccidials has also been reported [49, 50, 59].

Chemical anticoccidials and ionophores generally negatively affect the metabolism and ion transport, respectively, of the parasites [35]. However, some anticoccidials are not entirely efficient in all the developmental stages of *Eimeria* species [18]. The development of *Eimeria* species resistance to ionophores is linked to a change in the biochemical composition of the parasite membrane [8]. In the field, husbandry practices that include the intensive use of anticoccidials is often the cause of development of resistance. The use of anticoccidials in Korea has decreased significantly, from about 52,612 kg in 2014 to 23,682 kg in 2019. However, the poultry industry still uses anticoccidials such as clopidol, diclazuril, maduramycin, monensin, and salinomycin as prophylactic medications [60].

## Conclusion

Taken together, the results of this study indicated that the prevalence of coccidiosis in South Korea remains high, and that over time the population dynamics and infection levels of *Eimeria* species have changed. Furthermore, there are *Eimeria* species in the field with severe levels of multi-drug resistance to anticoccidials. Therefore, it is critical to look for alternative control methods that consider drug-sensitivity profiles of field samples found in this study. Periodic monitoring of resistance, field status, and prevalence of *Eimeria* species is critical, because knowing the patterns will be helpful for planning and implementation of an optimized control program.

## Methods

### Fecal sample collection and oocyst counting

Fecal samples were collected from 388 randomly selected poultry farms in Korea between December 2019 and May 2021. All sampled farms have no history with the use of coccidiosis vaccine in their programs. The 388 fecal samples were collected from 278 broiler farms, 61 laying hen farms, 27 native chicken farms, 12 broiler breeder farms and 10 laying breeder farms. Additionally, farms consisted of 75 cage and 313 free-ranged types of management (Table 1). Each sample consisted of a pool of fresh manure collected from several chickens or a pooled fecal sample from areas distributed in the litter of randomly selected poultry farm buildings. The collected samples were put into sealable plastic bags or screw-cap containers and were sent to the laboratory. One-gram fecal samples were homogenized in 5 ml saturated NaCl solution using a vortex mixer. All fecal samples were first examined for *Eimeria* oocysts using a standard McMaster technique, as previously described [20]. The number of oocysts was presented as the number of oocysts per gram of feces (OPG). The lower detection limit of the fecal examination was less than 50 OPG.

### Genomic DNA extraction from oocysts

Before genomic DNA was extracted from fecal oocysts, positive fecal samples were diluted fivefold with phosphate-buffered saline (PBS, pH 7.4), homogenized using a vortex mixer, and then filtered using a mesh sieve. The materials that did not pass through the sieve were discarded. The filtrate was then transferred to a plastic centrifuge container and centrifuged for 10 min at 1,000 × *g*. The supernatant was discarded, while the sediment containing the oocysts was resuspended with PBS and washed two times using centrifugation (1,000 × *g*, for 10 min). After washing, the sediment was resuspended with 2.5% potassium dichromate solution (Samchun Chemicals, Pyongtack, Korea) and incubated at 28 °C for 1–3 days with shaking and aeration to achieve sporulation. Oocysts sporulation was checked using microscopy at 100 × magnification. The samples were washed twice using PBS (pH 7.4) by centrifugation to remove the potassium dichromate solution. They were then resuspended in PBS. Genomic DNA extraction was performed using the QIAamp® Fast DNA Stool Mini Kit according to the manufacturer’s protocol (Qiagen, Germany).

### Molecular characterization of Eimeria species using PCR

*Eimeria* species was determined using PCR primers (Table 4) targeting the internal transcribed spacer 1 (ITS-1) of nuclear ribosomal DNA of each *Eimeria* species. Each PCR reaction was performed in a 20 μl PCR premix (Bioneer, South Korea) containing 1 μl genomic DNA template and 10 p mol each for the forward and reverse primers, as previously described [20]. The cycling program for the primary PCR using ITS-1 primers consisted of a denaturation step for 5 min at 95 °C, followed by 30 cycles at 95 °C for 45 s, 55 °C for 45 s, and 72 °C for 1 min, then a final extension of 72 °C for 4 min in a thermal cycler. The secondary PCR reaction with the primary PCR product was performed as previously described [61]. Briefly, initial denaturation was performed for 5 min at 95 °C, then 30 cycles at 95 °C for 30 s, 58 or 65 °C for 30 s, and 72 °C for 1 min, followed by a final extension at 72 °C for 5 min. Each PCR product was loaded to a 1.5% agarose gel with ethidium bromide and was run in TAE buffer for 25 min. The DNA fragments were visualized using ultraviolet light.

**Table 4:** Specific primers targeting the Internal Transcribed Spacer Region-1 (ITS-1) of different *Eimeria* species in chickens

Species	Primer sequences 5’-3’		Expected amplicon size (bp)	Annealing Temperature (°C)	References
ITS-1	F	AAGTTGCGTAAATAGAGCCCTC	variable	56	[62]
	R	AGACATCCATTGCTGAAAG			
*E. acervulina*	F	GGGCTTGGATGATGTTTGCTG	145	65	[61]
	R	GCAATGATGCTTGCACAGTCAGG			
*E. brunetti 1*	F	CTGGGGCTGCAGCGACAGGG	183	58	[62]
	R	ATCGATGGCCCCATCCCGCAT			
*E. brunetti 2*	F	GATCAGTTTGAGCAAACCTTGC	311	65	[61]
	R	TGGTCTTCCGTACGTCGGAT			
*E. maxima 1*	F	GTGGGACTGTGGTGATGGGG	205	65	
	R	ACCAGCATGCGCTCACAACCC			
*E. maxima 2*	F	TTGTGGGGCATATTGTTGTGA	162	60	
	R	CWCACCACTCACAATGAGGCAC			
*E. mitis*	F	GTTTATTTCCTGTCGTCGTCTCGC	330	65	
	R	GTATGCAAGAGAGAATCGGGATTCC			
*E. necatrix*	F	AGTATGGGCGTGAGCATGGAG	160	58	
	R	GATCAGTCTCATCATAATTCTCGCG			
*E. praecox*	F	CATCGGAATGGCTTTTTGAAAGCG	215	65	
	R	GCATGCGCTAACAACTCCCCTT			
*E. tenella*	F	AATTTAGTCCATCGCAACCCTTG	278	65	
	R	CGAGCGCTCTGCATACGACA			

### Anticoccidial drug sensitivity test

#### Experimental design and infection

The anticoccidial drug sensitivity test was carried out for a total of 16 days in an experimental animal facility in Gyeongsang National University. Day-old ROSS 308 female broiler chicks (Samhwa, Korea) were housed in cages in a coccidiosis-free environment and were given ad libitum access to water and feed throughout the experiment. To make similar and balanced groups before treatment, the weights of 5-day-old chicks were measured and the chicks weighing 110-125 g were randomly assigned to groups (*n* = 40/group): uninfected/untreated control group, infected/untreated groups and infected/treated groups. Treatment and infection was randomized to each group using = RANDBETWEEN () function in Microsoft Excel and the groups were randomly allocated to cages.

Anticoccidial drug sensitivity testing of nine field samples (Additional file 1) to the six anticoccidials clopidol (125 ppm), diclazuril (1 ppm), maduramycin (5 ppm), monensin (100 ppm), salinomycin (60 ppm), and toltrazuril (25 ppm) was performed. At 7 days old, the chicks were weighed and orally inoculated with 3 X 10^4^ sporulated oocysts of the field samples to their corresponding group and fed a standard diet supplemented with anticoccidial drugs beginning 2 days before infection and continued throughout the entire experimental period. Oocysts used for infection were cleaned using flotation on 5.25% sodium hypochlorite; they were then washed three times with PBS. The untreated/infected control birds (positive control, PC) were given their respective inoculums, while the uninfected untreated control birds (negative control, NC) were given PBS. A dose titration trial on the nine different field samples was performed to determine the inoculation dose to cause a weight gain reduction of 15–25% in infected and unmedicated birds [63].

Birds were observed daily and the mortality was monitored. The following parameters were measured: body weight gain, oocyst number/g and lesion scores. Body weight gain (*n* = 20) was measured each bird at 9 days post-infection. Fecal samples were collected from 6 to 9 days post-infection and were homogenized using a blade grinder. Two 20-ml samples were collected from each suspension. Samples were diluted in saturated NaCl, and oocysts were counted microscopically using a McMaster counting chamber. Total oocyst numbers were calculated using the formula: [total oocyst number = oocyst count × dilution factor × (fecal sample volume/counting chamber volume)/number of birds per cage]. Intestinal lesion score (*n* = 5, randomly picked) was evaluated from each group at 7 days post-infection, based on previously described scoring techniques [24]. The lesion score for each chicken was assigned a numerical value from 0 to 4 and was carried out blindly by three individuals.

#### Evaluation of drug resistance

Anticoccidial drug resistance of the *Eimeria* species in the farm samples was evaluated using the anticoccidial index (ACI), percent optimum anticoccidial activity (POAA), relative oocyst production (ROP), and reduced lesion score (RLS).

The ACI was calculated as previously described [42]: ACI = (rate of relative body weight gain + survival rate) – (lesion score + oocyst value). Oocyst value = (fecal oocyst number of treated and infected group ÷ fecal oocyst number of PC group) X 100. An ACI value ≥ 160 indicated sensitivity and an ACI value < 160 indicated resistance. POAA values were computed as previously described [42, 49]: POAA = [(average GSR of treated and infected group—average GSR of PC group) ÷ (average GSR of NC group—average GSR of PC group)] X 100. GSR = (total weight at end of experiment + weight of dead birds) ÷ total weight at start of experiment. A POAA value ≥ 50% indicated sensitivity and a POAA value < 50% indicated resistance. The ROP value was computed as previously described [49]: ROP = (oocyst output of treated and infected group ÷ oocyst output of PC group) X 100. An ROP value < 15% indicated sensitivity and a value ≥ 15% indicated resistance. RLS were computed as previously described [64] = (mean lesion score of PC group ÷ mean lesion score of treated and infected group)/mean lesion score of PC group X 100. An RLS > 50% indicated sensitivity and RLS ≤ 50% indicated resistance.

Each index was assigned a point system in the presence of resistance. The overall assessment of drug resistance was adapted from Lan et. al [49]. Briefly, field samples were considered to be severely resistant when all or three indexes showed resistance (3 or 4), moderately resistant when 2 of the indexes showed resistance, slightly drug resistant if 1 showed resistance, and no drug resistance (0) in the absence of indexes indicating resistance.

### Data analysis

Associations between risk factors and infection were evaluated using two-tailed Fisher’s exact tests or Chi-square (*X*^2^) tests. Body weight gain, lesion scores, and oocyst production were analyzed using Student’s *t*-tests or one-way ANOVA and Dunnett’s multiple comparison tests. All analyses were performed using Graphpad Prism version 9.0 and InStat statistical software (GraphPad, USA). Differences were considered statistically significant at *P* < 0.05. Results were expressed as mean ± standard error values.

## Supplementary Information

**Additional file 1. **Specific *Eimeria *species present in each farm sample.

**Additional file 2. **Survival rates of birds infected with farm samples.

**Additional file 3. **Anticoccidial Index (ACI) of each farm sample to different anticoccidials.

**Additional file 4. **Percent Optimum Anticoccidial Activity (POAA) of each farm sample to different anticoccidials.

**Additional file 5. **Relative Oocyst Production (ROP) of each farm sample to different anticoccidials.

**Additional file 6. **Reduction of Lesion Score (RLS) of each farm sample to different anticoccidials.

## Data Availability

The datasets used and/or analyzed during the current study are available from the corresponding author on reasonable request.

## Abbreviations

ACI: Anticoccidial Index
CI: Confidence Interval
ITS-1: Internal Transcribed Spacer 1
NC: Negative Control
NS: Not significant
OPG: The number of oocysts per gram of feces
OR: Odds Ratio
PBS: Phosphate-Buffered Saline
PC: Positive Control
POAA: Percent Optimum Anticoccidial Activity
RLS: Reduced Lesion Score
ROP: Relative Oocyst Production
