# Supplementary material for: Epidemiological investigation and drug resistance of Eimeria species in Korean chicken farms
Source: BMC Vet Res. 2022 Jul 14;18:277. doi: 10.1186/s12917-022-03369-3 (PMC9284840; doi:10.1186/s12917-022-03369-3)
Supplement: Supplementary file 1 — Additional file 1. Specific Eimeria species present in each farm sample. [file 12917_2022_3369_MOESM1_ESM.docx]

| **Additional file 1.** Specific *Eimeria* species present in each farm sample | | | | | |  |  |
| --- | --- | --- | --- | --- | --- | --- | --- |
| **Farm samples** | ***Eimeria* species** | | | | | | |
|  | *E. acervulina* | *E. maxima* | *E. tenella* | *E. necatrix* | *E. brunetti* | *E. praecox* | *E. mitis* |
|  |  |  |  |  |  |  |  |
| A | **+** | **+** | **+** |  |  | **+** |  |
| B | **+** | **+** | **+** |  |  | **+** | **+** |
| C | **+** | **+** | **+** |  |  | **+** | **+** |
| D | **+** |  | **+** |  |  |  | **+** |
| E | **+** | **+** | **+** |  |  | **+** | **+** |
| F | **+** | **+** | **+** |  |  | **+** | **+** |
| G | **+** | **+** | **+** |  |  |  |  |
| H | **+** | **+** | **+** | **+** | **+** |  | **+** |
| I | **+** | **+** | **+** |  |  |  | **+** |
| A-I, farm samples. | |  |  |  |  |  |  |
|  |  |  |  |  |  |  |  |
