# Supplementary material for: Epidemiological investigation and drug resistance of Eimeria species in Korean chicken farms
Source: BMC Vet Res. 2022 Jul 14;18:277. doi: 10.1186/s12917-022-03369-3 (PMC9284840; doi:10.1186/s12917-022-03369-3)
Supplement: Supplementary file 2 — Additional file 2. Survival rates of birds infected with farm samples. [file 12917_2022_3369_MOESM2_ESM.docx]

| **Additional file 2**. Survival rates of birds infected with farm samples | | | | | | | | | | |  |
| --- | --- | --- | --- | --- | --- | --- | --- | --- | --- | --- | --- |
| **Treatment** | **Farm samples** | | | | | | | | | |  |
|  | A | B | C | D | E | F | G | H | I | | |
| NC | 100 | 100 | 100 | 100 | 100 | 100 | 100 | 100 | 100 | | |
| PC | 90 | 100 | 90 | 100 | 90 | 100 | 90 | 100 | 90 | | |
| Clopidol | 100 | 100 | 100 | 90 | 100 | 100 | 100 | 90 | 100 | | |
| Diclazuril | 100 | 100 | 100 | 100 | 100 | 100 | 80 | 100 | 100 | | |
| Maduramycin | 100 | 100 | 100 | 100 | 90 | 100 | 100 | 100 | 100 | | |
| Monensin | 90 | 90 | 100 | 90 | 100 | 100 | 70 | 100 | 100 | | |
| Salinomycin | 100 | 100 | 90 | 100 | 100 | 100 | 90 | 100 | 90 | | |
| Toltrazuril | 100 | 100 | 100 | 100 | 100 | 100 | 100 | 100 | 100 | | |
| A-I, farm samples; NC, untreated and healthy chickens; PC, untreated and infected chickens. | | | | | | | | | |  |  |
