# Supplementary material for: Epidemiological investigation and drug resistance of Eimeria species in Korean chicken farms
Source: BMC Vet Res. 2022 Jul 14;18:277. doi: 10.1186/s12917-022-03369-3 (PMC9284840; doi:10.1186/s12917-022-03369-3)
Supplement: Supplementary file 3 — Additional file 3. Anticoccidial Index (ACI) of each farm sample to different anticoccidials. [file 12917_2022_3369_MOESM3_ESM.docx]

| **Additional file 3.** Anticoccidial Index (ACI) of each farm sample to different anticoccidials | | | | | | | | | | | |
| --- | --- | --- | --- | --- | --- | --- | --- | --- | --- | --- | --- |
| **Treatment** | **Farm samples** | | | | | | | | | | |
|  | A | B | C | D | E | F | G | H | I |  |  |
| NC | 200.00 | 200.00 | 200.00 | 200.00 | 200.00 | 200.00 | 200.00 | 200.00 | 200.00 |  |  |
| PC | 73.71 | 67.22 | 80.88 | 70.50 | 60.38 | 84.85 | 75.46 | 73.77 | 68.86 |  |  |
| Clopidol | 123.39 | 126.14 | 126.34 | 120.35 | 141.00 | 98.21 | 105.14 | 68.22 | 83.98 |  |  |
| Diclazuril | 122.08 | 115.15 | 114.47 | 105.65 | 150.74 | 79.49 | 82.34 | 131.73 | 75.32 |  |  |
| Maduramycin | 82.98 | 86.36 | 24.92 | 64.22 | 84.14 | 65.35 | 91.49 | 71.77 | 52.58 |  |  |
| Monensin | 107.72 | 102.63 | 111.68 | 93.96 | 98.51 | -4.41 | 49.70 | 54.80 | 55.26 |  |  |
| Salinomycin | 98.84 | 113.52 | 38.95 | 50.14 | 95.59 | 57.56 | 50.35 | 93.75 | 71.73 |  |  |
| Toltrazuril | 87.99 | 110.41 | 69.21 | 96.52 | 134.02 | 97.92 | 100.58 | 114.61 | 90.17 |  |  |
| Interpretation: ≥160, sensitive; ≤160, resistant; A-I, farm samples; NC, untreated and healthy chickens; PC, untreated and infected chickens. | | | | | | | | | | |  |
