# Supplementary material for: Epidemiological investigation and drug resistance of Eimeria species in Korean chicken farms
Source: BMC Vet Res. 2022 Jul 14;18:277. doi: 10.1186/s12917-022-03369-3 (PMC9284840; doi:10.1186/s12917-022-03369-3)
Supplement: Supplementary file 4 — Additional file 4. Percent Optimum Anticoccidial Activity (POAA) of each farm sample to different anticoccidials. [file 12917_2022_3369_MOESM4_ESM.docx]

| **Additional file 4.** Percent Optimum Anticoccidial Activity (POAA) of each farm sample to different anticoccidials | | | | | | | | | | |
| --- | --- | --- | --- | --- | --- | --- | --- | --- | --- | --- |
| **Treatment** | **Farm samples** | | | | | | | | | |
|  | **A** | **B** | **C** | **D** | **E** | **F** | **G** | **H** | **I** |  |
| NC | 100.00 | 100.00 | 100.00 | 100.00 | 100.00 | 100.00 | 100.00 | 100.00 | 100.00 |  |
| PC | 0.00 | 0.00 | 0.00 | 0.00 | 0.00 | 0.00 | 0.00 | 0.00 | 0.00 |  |
| Clopidol | 18.19 | 37.07 | 12.60 | 31.29 | 36.98 | 55.04 | 8.17 | 26.05 | 5.99 |  |
| Diclazuril | 3.65 | 16.19 | 17.32 | 43.62 | 32.92 | 13.02 | 34.97 | 28.45 | 1.34 |  |
| Maduramycin | 72.12 | 14.20 | 5.16 | 27.23 | 15.74 | 27.75 | 3.88 | 23.84 | 37.93 |  |
| Monensin | 25.59 | 10.23 | 7.29 | 28.45 | 27.80 | 36.62 | 165.99 | 47.14 | 54.10 |  |
| Salinomycin | 6.37 | 18.84 | 40.04 | 41.54 | 19.95 | 30.26 | 46.60 | 41.14 | 105.23 |  |
| Toltrazuril | 36.37 | 24.53 | 5.97 | 49.01 | 17.09 | 7.92 | 42.23 | 3.63 | 0.92 |  |
| Interpretation: ≥50, sensitive; ≤50, resistant; A-I, farm samples; NC, untreated and healthy chickens; PC, untreated and infected chickens. | | | | | | | | | | |
