# Supplementary material for: Epidemiological investigation and drug resistance of Eimeria species in Korean chicken farms
Source: BMC Vet Res. 2022 Jul 14;18:277. doi: 10.1186/s12917-022-03369-3 (PMC9284840; doi:10.1186/s12917-022-03369-3)
Supplement: Supplementary file 5 — Additional file 5. Relative Oocyst Production (ROP) of each farm sample to different anticoccidials. [file 12917_2022_3369_MOESM5_ESM.docx]

| **Additional file 5.** Relative Oocyst Production (ROP) of each farm sample to different anticoccidials | | | | | | | | | | |
| --- | --- | --- | --- | --- | --- | --- | --- | --- | --- | --- |
| **Treatment** | **Farm samples** | | | | | | | | | |
|  | **A** | **B** | **C** | **D** | **E** | **F** | **G** | **H** | **I** |  |
| NC | 0.00 | 0.00 | 0.00 | 0.00 | 0.00 | 0.00 | 0.00 | 0.00 | 0.00 |  |
| PC | 100.00 | 100.00 | 100.00 | 100.00 | 100.00 | 100.00 | 100.00 | 100.00 | 100.00 |  |
| Clopidol | 61.72 | 55.60 | 53.18 | 44.81 | 39.02 | 80.57 | 79.50 | 100.99 | 96.79 |  |
| Diclazuril | 62.54 | 56.24 | 69.98 | 74.02 | 28.30 | 99.63 | 81.11 | 46.92 | 104.78 |  |
| Maduramycin | 92.46 | 86.77 | 155.15 | 107.38 | 73.13 | 116.37 | 82.70 | 95.68 | 119.55 |  |
| Monensin | 61.14 | 50.64 | 69.14 | 83.26 | 67.49 | 185.34 | 80.55 | 106.87 | 116.77 |  |
| Salinomycin | 81.63 | 59.24 | 123.52 | 127.97 | 67.06 | 129.20 | 118.91 | 70.25 | 78.12 |  |
| Toltrazuril | 90.91 | 64.45 | 108.09 | 84.85 | 39.38 | 85.72 | 75.88 | 55.49 | 88.36 |  |
| Interpretation: ≤15, sensitive; ≥15, resistant; A-I, farm samples; NC, untreated and healthy chickens; PC, untreated and infected chickens. | | | | | | | | | | |
