# Supplementary material for: Epidemiological investigation and drug resistance of Eimeria species in Korean chicken farms
Source: BMC Vet Res. 2022 Jul 14;18:277. doi: 10.1186/s12917-022-03369-3 (PMC9284840; doi:10.1186/s12917-022-03369-3)
Supplement: Supplementary file 6 — Additional file 6. Reduction of Lesion Score (RLS) of each farm sample to different anticoccidials. [file 12917_2022_3369_MOESM6_ESM.docx]

| **Additional file 6.** Reduction of Lesion Score (RLS) of each farm sample to different anticoccidials | | | | | | | | | | |
| --- | --- | --- | --- | --- | --- | --- | --- | --- | --- | --- |
| **Treatment** | **Farm samples** | | | | | | | | | |
|  | **A** | **B** | **C** | **D** | **E** | **F** | **G** | **H** | **I** |  |
| NC | 100.00 | 100.00 | 100.00 | 100.00 | 100.00 | 100.00 | 100.00 | 100.00 | 100.00 |  |
| PC | 0.00 | 0.00 | 0.00 | 0.00 | 0.00 | 0.00 | 0.00 | 0.00 | 0.00 |  |
| Clopidol | -7.69 | 13.33 | 0.00 | -70.00 | 36.84 | 30.00 | 38.89 | 22.22 | 41.18 |  |
| Diclazuril | 23.08 | 6.67 | -8.33 | -60.00 | 31.58 | 40.00 | 27.78 | 11.11 | 5.88 |  |
| Maduramycin | 0.00 | 26.67 | -16.67 | -100.00 | 15.79 | 30.00 | 16.67 | 11.11 | -17.65 |  |
| Monensin | 7.69 | -6.67 | -33.33 | -20.00 | 31.58 | 30.00 | -11.11 | 0.00 | -5.88 |  |
| Salinomycin | 15.38 | -20.00 | 0.00 | -60.00 | 10.53 | 30.00 | 16.67 | -11.11 | 0.00 |  |
| Toltrazuril | 15.38 | 26.67 | 8.33 | -60.00 | 36.84 | -10.00 | 27.78 | -5.56 | 0.00 |  |
| Interpretation: >50, sensitive; ≤50, resistant; A-I, farm samples; NC, untreated and healthy chickens; PC, untreated and infected chickens. | | | | | | | | | | |
